# Immunogenicity, Pathogenesis, and Host’s Immuno-Responses to Marburg Virus Infection

**DOI:** 10.3390/pathogens14040323

**Published:** 2025-03-27

**Authors:** Emmanuel Edwar Siddig, Nicaise Ndembi, Ayman Ahmed, Claude Mambo Muvunyi

**Affiliations:** 1 Rwanda Biomedical Center (RBC), Kigali KG 644 St, Rwanda; emanwelleds389@gmail.com; 2 The Africa Centres for Disease Control and Prevention (Africa CDC), Ring Road, 16/17, Haile Garment Lafto Square, Addis Ababa P.O. Box 3243, Ethiopia; 3 The International Vaccine Institute (IVI), Africa Regional Office (IARO), Kigali KN 78 St, Rwanda; 4 Pan-Africa One Health Institute (PAOHI), Kigali KG 203 St, Rwanda

**Keywords:** hemorrhagic fever, viral dissemination, pathogenicity, immunogenicity, organs failure, clinical outcomes

## Abstract

Due to the sudden emergence and burnout nature of Marburg virus (MARV) outbreaks, little is known about MARV’s pathogenicity and immunogenicity. These gaps in knowledge are limiting our understanding of the disease and the implementation of cost-effective prevention and control measures including case management through safe and effective therapeutic modalities. Therefore, this review aims to synthesize and summarize evidence about pathogenicity, immunogenicity, and virulence in humans towards MARV. Upon infection, MARV rapidly disseminates throughout various tissues, provoking severe cellular injury, particularly in lymphatic organs, the liver, kidneys, and the gastrointestinal tract. The virus takes advantage of host cells by avoiding immune responses, mainly by disrupting the function of dendritic cells and blocking the signaling pathways for interferon. As a result, patients experience profound immune dysregulation characterized by early lymphocyte depletion and a shift towards pro-inflammatory cytokine release, resulting in a cytokine storm that can lead to hemorrhagic septic shock. Additionally, adaptive immune responses, including antibody production, are impaired, further complicating recovery and increasing susceptibility to severe disease outcomes. Understanding these intricate host–pathogen interactions is critical for developing effective therapeutic strategies and vaccines against MARV. Continuing research is essential to explain the mechanisms of immune evasion and to identify potential intervention points for improving patient outcomes.

## 1. Introduction

Marburg virus disease (MVD) is a highly fatal zoonotic hemorrhagic fever viral disease that is caused by one of two sibling RNA viruses, both belonging to the species *Orthomarburgvirus marburgense* under the genus *Orthomarburgvirus*; namely Marburg virus (MARV) and/or Ravn virus, from the viral family *Filoviridae* [1,2,3]. Characterized by a severe viral hemorrhagic fever that can lead to a high mortality rate of up to 90% [3]. Therefore, the disease is considered a biological threat to Global Health Security by negatively impacting human’s security, health, and socioeconomic stability and growth [4,5]. Recent modeling studies have projected that up to 27 countries hosting over 100 million individuals are at risk of MARV outbreaks, underscoring the urgency of improving our understanding of this virus to develop safe and cost-effective prevention, response, and case management tools [6]. Globally leading technical agencies of global health, including the World Health Organization (WHO) and the Global Alliance for Vaccines and Immunizations (GAVI), are particularly important in addressing these requirements [7,8,9].

This review aims to elucidate the intricate kinetic of MARV within the host-body system and the corresponding host immunological responses towards the virus. Exploring these critical interactions, we will improve our understanding of MARV pathogenesis and uncover pivotal opportunities for the development and implementation of therapeutic interventions. A thorough analysis of these immune dynamics not only sheds light on the complexities underlying MVD but also serves as a foundation for developing safe and effective case management strategies.

## 2. Overview of MARV Disease Progression

After an individual acquires MARV infection, the initial presentation typically includes flu-like symptoms such as fever, headache, sore throat, and muscle and joint pain [10]. These symptoms generally appear 2 to 21 days after infection [11]. Within the first five days, gastrointestinal symptoms may arise, including abdominal pain, diarrhea, nausea, and vomiting [11]. As the disease progresses, particularly between days 5 to 7, patients may experience a rapid escalation of symptoms [11]. This phase may involve the development of a rash, conjunctivitis, and the emergence of hemorrhagic fever symptoms, including bleeding from mucosal surfaces, melena, and hematemesis. Neurological symptoms can also manifest, which may include confusion, agitation, seizures, and potentially coma in the late stages of the disease [12,13]. At this advanced stage, the virus spreads to the kidneys and liver, indicated by elevated liver enzyme levels and increased serum creatinine, signaling damage to these organs. Throughout the first week of symptom development, patients typically exhibit a low lymphocyte count and decreased platelet levels [14].

The clinical course of MARV disease can lead to two outcomes: recovery or death [14]. Fatalities often result from dehydration, bleeding, organ failure, and an immune response dysregulation exacerbated by systemic factors. Survivors may experience lingering symptoms during or after recovery, including arthritis, conjunctivitis, myalgia, and psychosis [15]. Serum samples from recovered patients typically display an IgG response to non-structural protein (NP) and glycoprotein (GP). However, the neutralizing antibody titer tends to diminish over time, with notable declines beginning at 21 months post-infection, eventually dropping below detectable levels by 27 months [16].

## 3. Viral Entry and Budding

When someone comes into contact with the MARV through a needle or a cut in the skin, the virus starts to infect the host cells [17]. The process of the virus entering these cells happens in several important steps. First, the virus sticks to the surface of the cell. Then, the cell takes the virus inside through a process called endocytosis, and finally, the virus fuses with the cell’s membrane to enter. This attachment is facilitated by different methods, including clathrin-mediated endocytosis, macropinocytosis, and the virus’s glycoproteins helping it bind to cell receptors [18]. The virus needs to interact with various receptors on the cell, like tyrosine kinase receptors, C-type lectin receptors, and a specific receptor called Niemann-Pick C1-like 1 (NPC1), to successfully enter the cell [19].

Once attached, MARV can enter the host cell through endocytosis or budding [17]. Notably, the MARV genome consists of seven structural genes: NP, VP35, VP40, VP30, GP, VP24, and L, comprising a single-stranded negative-sense RNA that assembles to form viral particles [20]. The matrix protein VP40 is particularly important because it self-assembles to create virus-like particles. This assembly process is significantly influenced by the levels of myosin-10 and Cdc42—proteins that play critical roles in filopodia formation and function. Additionally, VP40 interacts with the viral nucleocapsid, providing a crucial interface for MARV subviral particles and filopodia. These filopodia facilitate contact with neighboring cells and enable the virus to spread effectively, resulting in high viral titers observed in the blood of infected host [21,22]. The release of viruses from the basolateral membranes of infected host cells allows access to underlying tissues and the vascular system, which can lead to severe infections. Both the basolateral aspects of hepatocytes and biliary epithelial cells are implicated in MARV budding, with the VP40 protein being essential for releasing infectious particles that promote disease progression [17,21].

## 4. How Marburg Virus Targets Cells: Receptor-Mediated Infection

**Figure 1:**
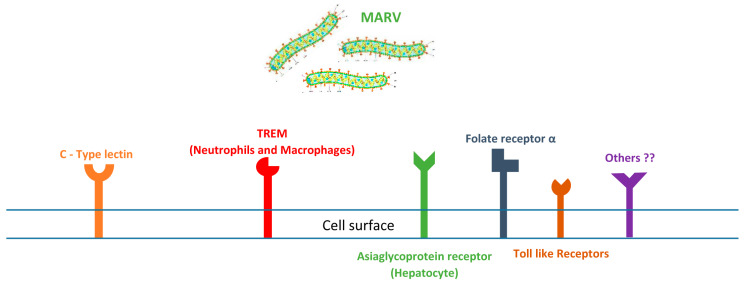
Interaction of MARV with the host’s cell also known as Recognition of MARV cellular receptors. It illustrates the binding of Marburg virus’s glycoproteins with the host (human) dendritic cells (DCs) during the virus entry to the cell to establish an infection.

MARV interacts with pathogen-associated molecular patterns (PAMPs) during infection, which play a critical role in the host immune response [23]. PAMPs are molecules associated with groups of pathogens, recognized by the immune system as indicators of infection [24,25]. In the case of MARV, certain PAMPs can trigger innate immune responses, leading to the production of interferons and other pro-inflammatory cytokines (Figure 1) [26,27]. However, MARV has evolved mechanisms to evade these immune responses, allowing it to replicate and spread [26]. For instance, the virus can inhibit the activation of dendritic cells and disrupt the signaling pathways that are initiated by PAMP recognition, which may contribute to its virulence and the severity of disease in infected individuals. Understanding the interplay between MARV and PAMPs is crucial for developing strategies aimed at enhancing immune responses during infection.

Studies have shown that the MARV primarily targets certain immune cells early in the infection, including dendritic cells (DCs), monocytes, macrophages and endothelial cells [28,29]. As the infection continues, MARV can also infect different types of cells in the body, not just those in the lymphatic system [30].

Several cellular receptors facilitate MARV infection, and those identified appear to be relatively nonspecific or pattern-based. For example, C-type lectins such as DC-specific intercellular adhesion molecule 3 (ICAM3)-grabbing non-integrin (DC-SIGN; CD209) and liver/lymph node-SIGN (L-SIGN; CLEC4M) are sufficient to enable MARV glycoprotein-mediated infection [31,32]. Additionally, the human macrophage C-type lectin that specifically recognizes galactose/N-acetylgalactosamine (hMGL), expressed by monocytic cells such as immature DCs, also enhances MARV entry [33]. The asialoglycoprotein receptor, found on hepatocytes, exhibits a particular affinity for the N-linked sugar chains with terminal galactose residues present in MARV glycoproteins, facilitating viral cell entry [31]. Interestingly, the binding of MARV to C-type lectins can be significantly inhibited by the carbohydrate mannan, suggesting that the interaction between the MARV glycoproteins and host cell surface carbohydrate motifs plays a crucial role in infection [34].

Recent findings indicate that a class of immunorecognition receptors known as TREM (triggering receptors expressed on myeloid cells) may have a significant early role in MARV interaction with immune cells like neutrophils, monocytes, and mature DCs [35,36]. These observations about the binding of MARV to C-type lectin and TREM receptors on myeloid lineage cells align with in vivo studies. However, MARV is pantropic and can infect other cell types, such as endothelial and epithelial cells, which do not express C-type lectins or TREMs. Other ubiquitous molecules, like heparin-sulfate proteoglycan (HSPG) and folate receptor-α may also facilitate viral entry in these cell types [37].

The complexity of MARV interactions with host cells is further amplified by the fact that MARV glycoproteins are glycosylated differently depending on the host cell type in which they are produced. Furthermore, MARV viral particles may bud from lipid rafts, potentially acquiring host proteins that influence viral tropism [38]. Notably, lactoferrin—an antimicrobial and immunoregulatory protein released by neutrophils upon MARV binding—has been observed to enhance the uptake of MARV by immature DCs. Additionally, toll-like receptors (TLRs) play a vital role in the ability of innate immune cells to detect pathogens and establish adaptive immune responses. Preliminary data suggest that TLR1 expression, which signals in conjunction with TLR2, is significantly increased on MARV-activated neutrophils, while the expression levels of other TLRs remain unaltered [39,40]. Continued research is necessary to ascertain the pathogenic significance of these early interactions between MARV and innate immune cells, as well as their potential as intervention targets in the disease process.

## 5. Cellular Injury and Viral Targeting

**Figure 2:**
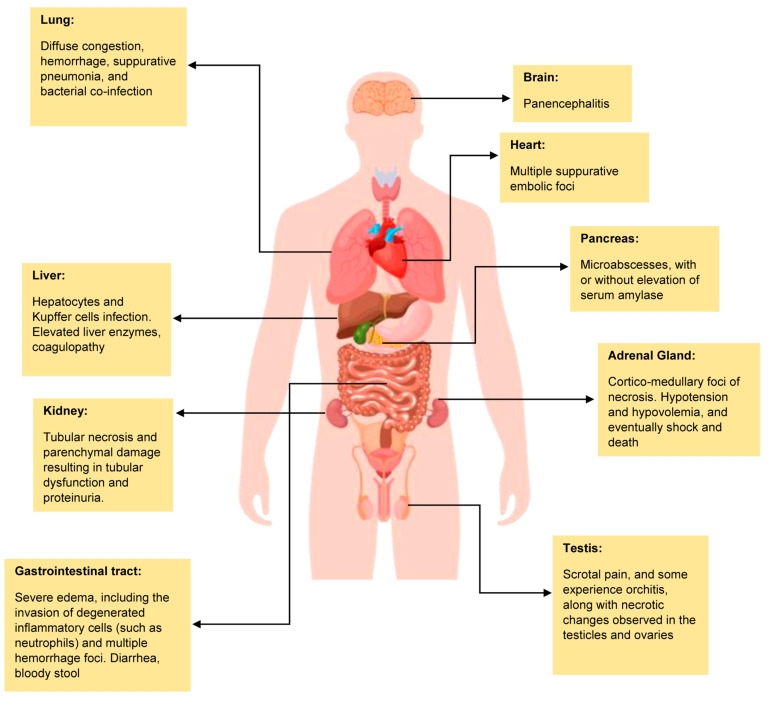
Summary of MARV manifestations in different organs of the human body.

Once the host becomes infected with MARV, the virus spreads quickly to many host cells [10]. Autopsy reports from infected hosts show that areas like the heart, brain, spleen, kidneys, and lymph nodes are significantly swollen. In non-human primates, bleeding can be seen in the mucous membranes and soft tissues, with the most severe damage found in the lymph nodes, liver, spleen, testes, ovaries, gastrointestinal tract, and the heart’s inner lining (Figure 2) [41]. These organs contain a high number of reticuloendothelial cells, which allow the virus to spread and cause increased blood vessel leakage and activate the blood clotting process. In the later stages of the disease, bleeding occurs in the gastrointestinal tract and in the cavities around the heart, lungs, and abdomen, often along with the buildup of fibrin.

### 5.1. Liver

Upon entering the liver, MARV interacts with the asialoglycoprotein receptor [33]. Following this, various histological changes are observed in hepatocytes and Kupffer cells [42]. Infected hepatocytes exhibit varying degrees of necrosis, with the extent of damage depending on the disease stage. Histopathological changes may demonstrate mild to moderate steatosis and hyperplasia of Kupffer cells [10]. The cytopathic effects of MARV in hepatocytes include intracellular eosinophilic inclusions, primarily found in periportal zones and areas surrounding necrosis [43]. Clinically, the infection is reflected in elevated liver enzymes such as alanine aminotransferase (ALT) and aspartate aminotransferase (AST), indicating hepatocellular damage [11,44,45,46]. In advanced stages of infection, severe liver injury can lead to coagulopathy, characterized by prolonged prothrombin time (PT) and partial thromboplastin time (PTT), which increases the likelihood of disseminated intravascular coagulation (DIC) and multi-organ failure [47].

### 5.2. Adrenal Gland

The involvement of the adrenal glands during MARV infection is marked by the presence of cortico-medullary foci of necrosis [48]. Additionally, infected adrenal cells show cytopathic effects, including eosinophilic intracellular inclusion bodies similar to those found in liver cells [42]. Consequently, patients develop hypotension and hypovolemia due to impaired steroid synthesis, potentially leading to shock and death [49].

### 5.3. Pancreas

As the infection spreads to the pancreas, patients may display microabscesses caused by bacterial emboli, although other pancreatic tissues might appear normal [42]. The pancreatic islet cells, particularly the beta cells, are the most affected. Approximately half of the islet cells contain viral inclusions, as confirmed by immunohistochemistry and electron microscopy. Infected beta cells are significantly more numerous than alpha cells. Some of the literature notes instances of pancreatitis associated with MARV, but they often do not specify the timing of these events [19]. Serum amylase concentrations reported range from normal to elevated [50], while lipase levels have not been extensively studied [45].

### 5.4. Spleen

When MARV infects the spleen, the virus initiates entry and replication within infected cells, leading to the disruption of the normal splenic architecture [51]. Moreover, when the infection reaches the lungs, the alveoli often exhibit diffuse congestion, hemorrhage, suppurative pneumonia, and bacterial co-infection. In patients infected with the MARV, small necrotic foci and micronecrosis are commonly identified in alveolar macrophages, while the endothelia of alveolar capillaries are frequently disturbed [42,52,53].

### 5.5. Gastrointestinal Tract

In terms of the gastrointestinal tract, a significant presence of plasma cells and monocytes can be observed within the lymphatic organs and mucous membranes of the stomach and intestines in non-human primates (NHPs) [51]. In humans, severe edema is noted in the submucosa, characterized by the infiltration of degenerated inflammatory cells, such as neutrophils, along with multiple foci of hemorrhage. Autolysis of intestinal tissues complicates cellular identification. Mild focal mononuclear penetration occurs in the lamina propria of the gastric, small intestinal, and colonic mucosa, with macrophages showing Marburg-like inclusions. The presence of virions in reticular fibrils and debris from necrotic cells elucidates the mechanisms behind human-to-human transmissions that may arise from exposure to bloody stools [51].

### 5.6. Kidney

The kidneys in MARV-infected patients present as swollen, pale, and hemorrhagic, with tubular necrosis and parenchymal damage resulting in tubular dysfunction and proteinuria. Multiple suppurative embolic foci associated with Gram-negative bacteria have been observed [54]. A viral antigen is found multifocally in the glomerulus and proximal tubular epithelial cells, as well as in interstitial connective tissues near capillaries. Marburg virus-like inclusions are seen in intertubular tissue macrophages and fibroblast-like cells, while some virions are noted in glomerular capillaries, although no viral antigen is detected in the medulla [51].

### 5.7. Skin and Mucous Membrane

In cases of hemorrhagic disease caused by the MARV, skin and mucous membranes often show bleeding abnormalities, resulting in skin lesions [55,56]. While there are few histopathological changes in the skin, common findings include swelling of endothelial cells, localized bleeding, tissue death, and fluid buildup in the skin. These skin changes usually appear between the second and seventh days after symptoms begin, and they may come back during the recovery phase. Viral antigens can be found in various skin cells, including epidermal dendritic cells, endothelial cells, connective tissue fibroblasts, and the cells in sebaceous and sweat glands [55].

### 5.8. Reproductive System

Furthermore, studies shows that the MARV can remain in semen for up to seven weeks after symptoms first appear, indicating a risk of sexual transmission [57,58,59]. Viral antigens have been found in the seminiferous tubules, which raises concerns about this possibility. Many patients report scrotal pain, and some experience orchitis, along with necrotic changes observed in the testicles and ovaries of those with Marburg hemorrhagic fever (MHF) [44]. In non-human primate survivors, ongoing MARV infections in testicular tissues can lead to serious testicular damage, including loss of sperm cells and inflammatory responses, particularly affecting Sertoli cells and disrupting the blood–testis barrier [60]. Additionally, the MARV infection can cause localized orchitis, destruction of germ cells, and substantial IgG antibody accumulation [60].

### 5.9. Bone Marrow

The specific changes in bone marrow due to MHF are not fully understood, but MARV antigens have been detected in normocellular bone marrow, leading to localized tissue necrosis. Notably, thrombocytopenia can occur without a simultaneous reduction in platelet production, similar to findings in Ebola virus infections [61].

### 5.10. Cardiovascular and Nervous System

In the heart and central nervous system, the morphological damage seen in MARV autopsy cases varies. Some cases have shown multiple suppurative embolic foci along with lesions containing Gram-negative bacteria, such as Pseudomonas, in the heart muscle, yet no viral antigens have been associated with these lesions. A few instances have revealed signs of panencephalitis, characterized by glial nodules and mild lymphocytic infiltration around blood vessels in the brain.

Regarding vascular changes, endothelial cells serve as primary targets for MARV replication, which is essential for sustaining and enhancing the viremic phase. Evidence of the virus budding from the apical plasma membrane supports this idea, while basolateral budding may facilitate the initial spread of the virus into adjacent tissues [62]. Since endothelial cells help maintain barriers between blood and surrounding tissues, their infection may lead to a breakdown of this barrier, facilitating further viral spread. The activation of endothelial cells and the subsequent release of inflammatory substances can increase blood vessel permeability, leading to DIC and eventually shock and bleeding [63].

## 6. Host Immune Response

Severe infection with MARV in humans is marked by immune suppression and delayed antibody responses [26]. Numerous animal models, including mice and macaques, have been established to investigate the pathogenesis of MARV.

### 6.1. Innate Immunity and MARV

Understanding the role of early innate immune activation is crucial for grasping the pathogenesis of MARV infections. An effective antiviral response relies on initial events that ensure a proper balance between activation and inhibitory signals in relation to antigen detection. Early innate immunity can be conceptualized in two ways: one being a broadly applicable response that includes pattern-recognition molecules, pro-inflammatory cytokines, and antiviral cell activities; the other being a more systematic gearing up for an antigen-specific response involving T cells and antibodies [64]. In the case of MARV, significant disturbances are observed during these initial immune reactions.

MARV has been shown to impair aspects of the host interferon (IFN) pathway. Key viral proteins, such as VP35, VP24 and VP40, are involved in effective evasion of immune responses [65,66]. Specifically, VP35 inhibits the production of type I IFNs (e.g., IFNα and IFNβ), while VP24 interferes with the signaling pathways of these interferons, as well as IFNγ, which are crucial for establishing an antiviral state in infected cells [23,65,67]. The antagonism of IFN responses has significant implications, contributing not only to elevated viral loads during later stages of the disease but also to the early dysregulation of innate immunity and very important for viral replication and pathogenesis. Interestingly, while the overall IFN response is not completely ablated—in fact, elevated levels of IFN can frequently be detected in the bloodstream during acute infections—MARV’s ability to evade immune activation creates a complex immune landscape.

Dendritic cells (DCs) serve a pivotal role in T-cell responses and T-cell-dependent antibody responses as antigen-presenting cells (APCs) [68,69]. In MARV-infected human DCs, an early dysfunction is observed—a failure to transition from the immature to mature antigen-presenting DC stage [70]. This impairment leads to reduced production of essential pro-inflammatory cytokines necessary for effective T-cell activation such as alpha interferon (IFN-α), interleukin 1B (IL-1B), IL-6, IL-10, IL-8, and IL-12 [70]. In comparison, while monocytes and macrophages can also be productively infected by MARV, they may respond differently by producing pro-inflammatory cytokines like TNF, despite their incapacity to produce IFNs [23]. This indicates a compromising effect on the co-stimulatory functions of infected DCs, which affects their ability to stimulate allogeneic T-cell responses.

**Figure 3:**
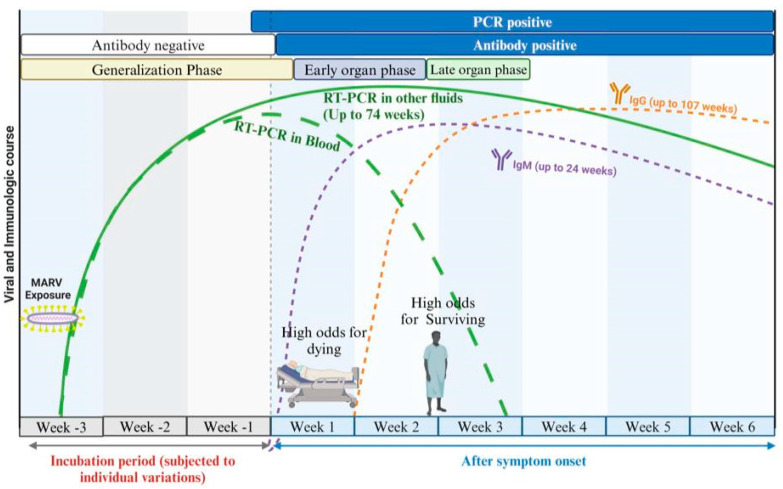
Illustrates the antibody response to MARV infection. IgM antibodies can appear within 4 to 7 days of symptom onset and may disappear within up to 6 months. Specific IgG antibodies typically develop 6 to 8 days after symptoms begin and can persist for several years. Additionally, RT–PCR is capable of detecting the virus generally 3 to 10 days post-symptom onset; however, it is important to confirm the clearance of the virus from the blood as well.

While the adaptive immune response, including the production of antibodies, is critical in combating MARV infection, it is significantly influenced by the early innate immune dysregulation. Antibodies play a crucial role in neutralizing viral particles and marking them for destruction by other components of the immune system [71,72]. Evidence from human sera indicates that immunoglobulin M (IgM) typically appears early in the immune response, often within the first week of infection (4 to 7 days). IgM serves as an initial defense mechanism, with titers peaking 1 to 2 weeks after the onset of the illness. Following this peak, IgM levels begin to decline over the next 1 to 2 months and disappear 30–168 days after infection. In contrast, immunoglobulin G (IgG) responses may emerge concurrently with (8–10 days) or after IgM but are associated with long-term immunity. IgG can often be detected several weeks after the initial infection, providing lasting protection against future encounters with the virus (Figure 3) [73,74].

Moreover, a study was conducted in order to investigate the humoral immune response to MARV infection by analyzing serum samples from uninfected controls and survivors. All survivors developed IgG responses to irradiated MARV antigens, with end titers ranging from 4.25 to 6 (LOG10 serum dilutions). Specific antibody responses were observed against the MARV nucleoprotein (NP) and glycoprotein (GP); however, survivors did not exhibit IgG reactivity to the VP35 or VP24 proteins. Some survivors had antibodies against VP40, while others responded to VP30. Additionally, neutralization tests revealed that only two survivors could neutralize MARV effectively in vitro, demonstrating positive neutralization responses at a threshold of ≥50% for samples collected 9 and 15 months post-infection. While the neutralizing antibody titers began to decline after 21 months, overall antibody levels against irradiated MARV remained stable over time. In comparison to Sudan virus (SUDV) survivors, who show long-lasting neutralizing antibody responses, MARV survivors exhibited more variability in their neutralizing responses. Despite a small sample size, it was noted that MARV survivors had lower overall neutralizing titers compared to SUDV survivors, who often had higher and more consistent neutralizing responses [16].

Understanding the antibody response is crucial for the diagnosis and surveillance of the MARV infection, as it provides valuable insights for healthcare professionals to monitor the immune response and assess disease outcomes. By analyzing antibody levels, particularly immunoglobulin G (IgG) and neutralizing antibodies, we can gain a clearer understanding of a patient’s recovery course and potential immunity to future infection. This information can facilitate targeted interventions. Moreover, knowledge of the variability in neutralizing responses among survivors underscores the importance of ongoing research and monitoring to determine the effectiveness of vaccinations and treatments, ultimately improving patient care.

Natural killer (NK) cells may also play an important role in the early defense against MARV. However, their effectiveness may be compromised as a result of impaired IFN production triggered by viral infection, highlighting species-specific differences in the susceptibility to interferon antagonism [75]. Furthermore, a study using non-human primates (*Cynomolus macaques*) demonstrated that after the inoculation of NHP with MARV Ci67 strain, the number of NK cells declined in the blood after the infection from 15% on the first day of infection to 5% on day 6 post-infection [76].

In addition to dendritic cells (DCs) and natural killer (NK) cells, MARV effectively replicates in monocytes, macrophages, and endothelial cells [70,77]. This persistence is supported by MARV’s ability to produce proteins that inhibit interferon (IFN) activity while promoting the release of pro-inflammatory cytokines. Immune cells infected by MARV can generate and secrete various cytokines, including IL-6, IL-8, and growth-regulated oncogene alpha (Gro-alpha/CXCL1) [78]. One hypothesis regarding the severe pathogenesis associated with MARV infection suggests that the excessive activation of immune cells leads to a significant cytokine release, resulting in a cytokine storm [26]. This cascade of events may induce a severe response akin to hemorrhagic septic shock. While this phenomenon is well documented in Ebola virus (EBOV), evidence specific to MARV remains limited.

Moreover, one study indicated that MARV strain Ci67 is associated with a rapid spike in pro-inflammatory cytokines observed at the time of death [76,77]. In the case of MARV Angola, a study involving *Cynomolgus macaques* demonstrated elevated levels of CCL2, CCL4, interleukin-6 (IL-6), and CXCL8 by days 7 to 9 post-aerosol challenge [79]. Furthermore, in rhesus macaques infected with MARV Angola, levels of IL-6 and monocyte chemotactic protein 1 (MCP-1) increased on day 7 before decreasing on day 8 [80]. Clearly, more research is needed to elucidate the immune response involved in MARV infections overall, as well as in MARV Angola specifically.

Additionally, viral particles have the ability to stimulate polymorphonuclear leukocytes, such as neutrophils, resulting in the release of inflammatory mediators from their granules. While these initial inflammatory responses can bolster the immune defense, they might also disrupt the functioning of other immune cells, especially dendritic cells [35].

The role of various cellular proteases, such as cathepsins, furins, and sheddases, warrants further investigation in the context of MARV infections. These enzymes are known to influence both the entry and exit mechanisms of viruses, including Ebola, shaping their tropism and interactions with host antibodies [81,82,83]. Recent research indicates that endosomal proteolytic enzymes, particularly cathepsins, are released from cells infected with Ebola virus, with heightened release observed in more virulent strains. This finding suggests that these proteases may significantly contribute to the virus’s pathogenesis, potentially facilitating vascular leakage and impacting innate immune responses. Understanding how these enzymes interact with the immune system could provide valuable insights into the mechanisms underlying MARV infections and the overall host response.

### 6.2. Adaptive Immune Response

In the context of MARV infection, DCs play a critical role in initiating adaptive immunity. However, the functionality of these cells is significantly compromised [84]. In normal circumstances, activated DCs promote optimal T cell activation, but MARV can manipulate these cells, leading to their dysfunction [76,84]. Specific co-stimulatory molecules, such as CD40, CD86, and IL-12, are often downregulated in infected DCs, preventing them from delivering necessary positive signals to T cells [70,85]. This disruption affects the sequential phases of the adaptive immune response—activation, antigen-specific expansion, contraction, and memory formation—making the development of effective vaccines more challenging [86].

The influence of MARV on DCs and monocytes appears to extend beyond a deficiency in positive signaling; it likely involves active co-inhibition of the immune response. Clinical evidence from humans and non-human primates infected with MARV reflects substantial apoptosis among lymphocyte populations in the peripheral blood and lymph nodes [76,87,88]. Although lymphocyte contraction is a standard process, the degree of depletion observed during MARV infections suggests a more severe disruption to immune homeostasis.

Numerous studies utilizing animal models, including mouse species [89], *Cynomolgus macaques* [76] and *Rhesus macaques* [80], have demonstrated that there is a significant loss of lymphocytes during the early stages of MARV infection. However, in the later stages, an increase in double positive (CD4 and CD8) T cells has been observed in macaques models [79].

Moreover, in general, following viral infection, T-cell co-signaling pathways are typically activated, involving interactions between CD28 on T cells and B7-family ligands, such as CD80 and CD86, on DCs [90]. However, these pathways can be exploited by co-inhibitory molecules like B7-H1 (PD-L1) [91]. This binding with PD1 on activated T and B cells can lead to T-cell silencing. The overexpression of B7-H1 on DCs and monocytes may significantly contribute to the dysregulation of T-cell responses. When PD1 engages with its ligand B7-H1, it transmits negative signals, exacerbating T-cell exhaustion and ultimately leading to their apoptosis [92,93]. The parallels seen in chronic infections underscore the potential relevance of B7-H1 and PD1 interactions in the immune dysfunction associated with viral infection [94].

Recent studies indicate that populations of CD8+ T cells are progressively diminished during MARV infection [76,87]. The lack of a robust immune response prior to the onset of severe disease further highlights the role of DC dysfunction, exemplified by their inability to properly regulate co-stimulatory molecules and produce essential cytokines in response to the MARV. Furthermore, in MARV infection, the cytokine IFNγ is secreted abundantly; yet, its role may be harmful. While it can have regulatory effects early in the immune response, excessive levels of IFNγ later in the infection could lead to pro-apoptotic effects on T cells, promoting further contraction and apoptosis [95]. Alongside IFNγ, other regulatory and inflammatory cytokines such as IL-10, TGF-β1, and IL-17, which are elevated during the initial stages of MARV infection, could further exacerbate immune dysregulation [26].

Additionally, MARV also impacts natural killer (NK) cells and invariant NKT (iNKT) cells [87]. The disruption of these cell populations can lead to increased inflammation and decreased antiviral responses. Co-inhibitory signals received by NK and iNKT cells may initiate a cascade of events that further contribute to immune dysfunction and cytotoxicity impairment. In general, the disruption of various adaptive immune cells during MARV infection illustrates the complexity of the immune response and its dysregulation. While DCs have been shown to suffer from compromised functionality—resulting in impaired T-cell activation and support—NK cells and iNKT cells also face significant challenges. NK cells, known for their rapid response to viral infections, demonstrate diminished cytotoxic activity due to the presence of co-inhibitory signals, further exacerbating the impact of MARV on the immune landscape. Invariant NKT cells play a role in bridging innate and adaptive immunity; however, their limited engagement and potential dysfunction in MARV infection hinder their ability to contribute effectively to antiviral responses. The interplay between these cell types, characterized by altered signaling and cytokine profiles, ultimately leads to a broader immune dysregulation, culminating in increased inflammation and reduced pathogen clearance. A comprehensive understanding of these mechanisms can inform future strategies aimed at restoring immune function and developing effective vaccines against MARV.

## 7. Conclusions

In summary, ongoing research continues to illuminate the multifaceted immune dysregulation associated with MARV infections. The pressing need for therapeutic strategies aimed at restoring immune balance is evident. Gaining a deeper understanding of the complex interactions between MARV and immune pathways is crucial for the development of targeted interventions that enhance outcomes for affected individuals. Future studies will be vital in clarifying the mechanisms governing these immune responses and establishing effective treatment approaches.

## Data Availability

All data produced during this study are included in the published article.
